# Changes in the ability to participate in and satisfaction with social roles and activities in patients in outpatient rehabilitation

**DOI:** 10.1186/s41687-020-00236-3

**Published:** 2020-09-01

**Authors:** Sietske J. Tamminga, Félicie M. van Vree, Gerard Volker, Leo D. Roorda, Caroline B. Terwee, Paulien H. Goossens, Thea P. M. Vliet Vlieland

**Affiliations:** 1 Basalt Rehabilitation, Leiden/The Hague, the Netherlands; 2 Department of Orthopaedics, Rehabilitation and Physical Therapy, Leiden University Medical Center, Leiden, the Netherlands; 3 Amsterdam Rehabilitation Research Center, Amsterdam, the Netherlands; 4 Department of Epidemiology and Biostatistics, Amsterdam UMC, Vrije Universiteit Amsterdam, Amsterdam, the Netherlands; 5 Merem Rehabilitation, Lelystad, the Netherlands

**Keywords:** Rehabilitation, Social roles and activities, Participation, Patient reported outcome measures, PROMIS

## Abstract

**Background:**

One of the main aims of rehabilitation is to improve participation. Patient-Reported Outcomes Measurement Information System (PROMIS®) item banks ‘Ability to Participate in Social roles and Activities, (PROMIS-APS) and ‘Satisfaction with Social Roles and Activities’ (PROMIS-SPS) are promising options to measure participation, but the literature on PROMIS measures of (satisfaction with) participation across diagnoses in rehabilitation is limited. Therefore, the objective of this study was to describe levels of and changes in participation, as assessed with the PROMIS-APS and the PROMIS-SPS short forms, of patients in outpatient rehabilitation.

**Methods:**

This study had quantitative, observational design with assessments at admission and discharge. Consecutive patients treated between April and August 2018 receiving outpatient multidisciplinary rehabilitation were the population of this study. The following diagnosis categories were included: brain injury (e.g. stroke), spinal cord and nerve injury, neuromuscular disorder (e.g. lateral sclerosis), amputation, musculoskeletal condition (e.g. osteoarthritis) or heart or lung disease (e.g. myocardial infarction, chronic obstructive pulmonary disease). The main patient-reported outcomes (PRO) of this study were the short form of the PROMIS-APS (8 items, Dutch general population reference score 50.6 [SD 9.5]), and PROMIS-SPS (8 items, Dutch general population reference score 47.5 [SD 8.3].

**Results:**

Of the 1279 patients invited, 777 (61%) completed the online forms at admission. Of those, 329 patients were invited at discharge, with 209 (64%) completing the forms. The mean (SD) T-scores of the PROMIS-APS and PROMIS-SPS were lower at admission (42.7 [SD 7.4]; (41.4 [SD 7.7]) and discharge (43.6 [SD 7.2]; (43.7 [SD 7.8]) than the Dutch general population. The change scores of the PROMIS-APS and PROMIS-SPS were 1.2 (95% CI 0.4–1.9; *p* = 0.004; effect size 0.16), and 2.4 (95% CI 1.6–3.2; *p* < 0.0001; effect size 0.31), respectively. In all diagnostic subgroups with > 30 paired measurements statistically significant improvements of PROMIS-APS, PROMIS-SPS or both were seen.

**Conclusions:**

Patients undergoing outpatient rehabilitation had, both at admission and discharge, considerably lower PROMIS-APS and PROMIS-SPS T-scores short forms than the general Dutch population, and showed small T-score improvements at discharge.

## Background

One of the primary aims of outpatient rehabilitation is improving activities of daily living and participation of patients with disabilities [1]. Personal goals of patients are often not only formulated in terms of alleviating impairments or limitations of activities, but in improving participation as well [2, 3]. In rehabilitation, activities and participation are often conceptualised according to the International Classification of Functioning Disability and Health (ICF) of the World Health Organisation (WHO) [4]. In that classification, participation is defined as “Involvement in a life situation”. Studies have shown that participation is strongly related to health related quality of life and well-being [5, 6].

To compare the effectiveness of rehabilitation across conditions or across rehabilitation centres or to relate the outcomes of rehabilitation to its costs (i.e. value based healthcare principles [7]), measurement instruments for participation should have adequate measurement properties. That is, these measurement instruments should be reliable, valid, responsive and change scores should be interpretable. Determining such properties of a measurement instrument for participation is however not easy, as previous research showed that participation is a difficult construct [8]. It encompasses a diversity of subdomains, such as paid work, sports or recreational activities, that might not be applicable or relevant to every individual subject [9].

Promising new methods to measure participation are instruments based on Item Response Theory (IRT) as they can be applied as short forms or Computer Adaptive Tests (CATs), resulting in less completion burden for patients. For participation this is for instance constituted by the IRT-based Patient-Reported Outcomes Measurement Information System (PROMIS®) v2.0 item bank ‘Ability to Participate in Social Roles and Activities’ (PROMIS-APS) and the v2.0 item bank ‘Satisfaction with Social Roles and Activities’ (PROMIS-SPS). A primary assumption of IRT is that the construct is unidimensional. Despite the above-mentioned challenges with the construct of participation, previous research found that the PROMIS-APS and PROMIS-SPS, show sufficient measurement properties including unidimensionality and reference scores have been determined recently [10, 11].

Although there is experience with PROMIS measures in rehabilitation (e.g. [12, 13]), the literature on PROMIS measures of (satisfaction with) participation across diagnoses in rehabilitation is limited. Therefore, the objective of this study was to describe levels of and changes in participation, as assessed with the PROMIS-APS and the PROMIS-SPS short forms, of patients in outpatient rehabilitation. Our hypothesis was that patients starting outpatient rehabilitation would have lower levels of participation compared to the general population and that they would improve considerably after outpatient rehabilitation.

## Methods

### Study design and setting

This observational study, used data that were routinely gathered in the context of care provided in a rehabilitation centre with two locations. The need for full medical ethical review and informed consent was waived according to Dutch Law as it concerned the voluntary completion of a concise questionnaire embedded in routine care [14]. The study was carried out in accordance with Dutch legislation regarding the storage and processing of patients’ personal data and in accordance with the Declaration of Helsinki [15].

All consecutive patients starting outpatient rehabilitation between April 1st and August 31st 2018 received an invitation by e-mail to complete an online questionnaire at admission and discharge. No reminders were sent.

Demographic and clinical information was derived from the clinic’s registry and medical records. The diagnosis was categorized as follows: brain injury (e.g. stroke, traumatic brain injury), spinal cord and nerve injury, neuromuscular disorder (e.g. lateral sclerosis), amputation, musculoskeletal condition (e.g. osteoarthritis) or heart or lung disease (e.g. myocardial infarction, chronic obstructive pulmonary disease).

### Daily clinical outpatient rehabilitation

In the current study, patients received multi-disciplinary outpatient rehabilitation in a large rehabilitation centre in the south-west part of the Netherlands, which is an urban area. By 2020 there are 18 medical specialist rehabilitation centres in the Netherlands, offering inpatient and outpatient rehabilitation [16]. Rehabilitation is accessible only after referral by a physician. Important criteria for admission include the need for multidisciplinary care and a potential for improvement in terms of return to society. Outpatient specialist rehabilitation includes, apart from medical care by a rehabilitation physician, care provided by the members of multidisciplinary team (consisting of physical and occupational therapists, social workers and psychologists) as required by the patient. Medical specialist rehabilitation treatment is included in the general medical insurance, and thus fully reimbursed.

### Patient-reported outcome (PRO)

#### Social participation and satisfaction with participation

The main PROs of this study were the short forms of the PROMIS-APS and PROMIS-SPS item banks [10, 11]. The PROMIS-APS measures “the perceived ability to perform one’s usual social roles and activities”. All items refer to limitations. The PROMIS-SPS measures “satisfaction with performing one’s usual social roles and activities”. Both short forms consists of 8 items and response options are provided on a 5-point Likert scale. They do not refer to a time frame. A higher score represents better ability to participate or more satisfaction. Good measurement properties were reported for both short forms [10]. The general Dutch population had PROMIS-APS and PROMIS-SPS T-scores of 50.6 (standard deviation (SD) 9.5) and 47.5 (SD 8.3) respectively, which were used as reference scores in this study [10].

### Statistical analysis

In order to determine the presence of selection bias with regard to those patients who did and did not complete all questionnaires, we compared the following characteristics of (1) patients who completed the measures both at admission and at discharge and patients (2) who completed the admission measures only: age, sex, diagnosis category, length of treatment, PROMIS-APS and PROMIS-SPS. Comparisons were done by means of Fisher’s Exact Test, Independent Samples T-test or Mann-Whitney U Test, where appropriate.

Mean changes between admission and discharge were computed with the 95% confidence interval with the paired Samples T-test or Wilcoxon Signed rank test, where appropriate. The effect size of the mean score difference was computed with Hedges’ gav with thresholds for small being 0.2, for moderate 0.5 and large for 0.8. Hedges’ gav is largely based on Cohen’s *d* but less biased leading to smaller effect sizes as it corrects for the fact that the measurements before and after rehabilitation are correlated within subjects [17, 18].

The mean changes between admission and discharge as well as the effect size were also carried out per diagnostic category if ≥30 paired measurements were available. Calculations of effect sizes and 95% confidence interval around effect sizes were performed using Excel [17]. All other analyses were done with SPSS Statistics, Version 22.0 [19].

## Results

Of the 1279 patients invited, 777 completed the PROMIS-APS and PROMIS-SPS at admission (61% response rate). Of the 329 patients invited at discharge, 209 completed both short forms at discharge (64% response rate). Patients who completed both measurements were significantly older, had more often a musculoskeletal condition and longer treatment duration (Table 1), but they did not differ statistically significant with respect to PROMIS-APS and PROMIS-SPS admission scores (data not shown).

**Table 1:** Sociodemographic and disease characteristics and duration of treatment of patients

		All patients *N* = 777	Patients completing admission assessment only *N* = 568	Patients completing both the admission and discharge assessments *N* = 209
Age in years; mean ± SD (range)		55 ± 15 (18–89)	55 ± 15 (18–89)	58 ± 15 (21–85)
Sex, male; No (%)		386 (50%)	286 (50%)	100 (48%)
Diagnosis; No (%)	Brain injury (e.g. stroke, traumatic brain injury)	392 (50%)	279 (49%)	113 (54%)
	Spinal cord and nerve injury	115 (15%)	89 (16%)	26 (12%)
	Neuromuscular disorder (e.g. lateral sclerosis)	107 (14%)	84 (15%)	23 (11%)
	Amputation	29 (4%)	25 (4%)	4 (2%)
	Musculoskeletal condition (e.g. osteoarthritis)	81 (10%)	49 (9%)	32 (15%)
	Heart or lung disease (e.g. myocardial infarction, chronic obstructive pulmonary disease).	53 (7%)	11 (7%)	11 (5%)
Rehabilitation duration; median (interquartile range)		121 (68–175)	115 (60–175)	126 (87–175)

The mean admission T-scores of the PROMIS-APS and the PROMIS SPS were 42.7 (SD 7.4) and 41.4 (SD 7.7), respectively, and about 1 SD below the general Dutch population reference T-scores [10]. The mean discharge T-scores of the PROMIS-APS and PROMIS-SPS were 43.6 (SD 7.2) and 43.3 (SD 7.8), respectively, and also below the general Dutch population reference T-scores.

The change in T-score on the PROMIS-APS was 1.2 (95% CI 0.4–1.9; *p* = 0.004) with an effect size of 0.16, and the change score on the PROMIS-SPS was 2.4 (95% CI 1.6–3.2; *p* < 0.0001) with an effect size of 0.31 (Table 2).

**Table 2:** PROMIS-APS and PROMIS-SPS T-scores at admission and discharge and change scores (T1 T0)

	PROMIS-APS (N = 209)^**1**^						PROMIS-SPS (***N*** = 205)^**1**^					
	T0 Mean ± SD	T1 Mean ± SD	∆ T1-T0 (95% CI)	n-pairs	***p***-value (T1 vs T0)	Effect size (T1 vs T0)	T0 Mean ± SD^**1**^	T1 Mean ± SD	∆ T1-T0 (95% CI)	npairs	***p***-value (T1 vs T0)	Effect size (T1 vs T0)
Total sample	42.4 ± 7.2	43.6 ± 7.2	1.2 (0.4–1.9)	209	0.004	0.16	41.1 ± 7.3	43.3 ± 7.8	2.4 (1.6–3.2)	205	< 0.0001	0.31
Brain injury	42.7 ± 6.7	44.0 ± 7.5	1.2 (0.2–2.2)	113	0.016	0.17	41.3 ± 7.3	43.9 ± 7.5	2.5 (1.5–3.6)	111	< 0.0001	0.34
Spinal cord and nerve injury	42.5 ± 8.1	42.5 ± 6.8	−0.02 (−2.5–2.4)	26	0.987	NA^2^	41.0 ± 7.0	43.0 ± 7.3	2.0 (−0.5–4.5)	26	0.115	NA^2^
Neuromuscular disorder	42.6 ± 9.8	42.9 ± 7.6	0.3 (−2.5–3.2)	23	0.812	NA^2^	40.1 ± 9.0	42.9 ± 7.5	2.8 (0.3–5.3)	22	0.010	NA^2^
Amputation	50.1 ± 4.3	45.3 ± 6.1	−4.8 (−18.8–9.3)	4	0.273	NA^2^	50.4 ± 1.4	47.0 ± 7.0	−3.4 (−15.5–8.7)	4	0.593	NA^2^
Musculoskeletal condition	40.6 ± 5.7	42.8 ± 6.4	2.2 (0.5–4.0)	32	0.012	0.37	39.4 ± 6.4	41.8 ± 9.3	2.5 (− 0.02–4.95)	31	0.064	NA^3^
Heart or lung disease	41.5 ± 7.5	45.3 ± 7.1	3.8 (−1.6–9.2)	11	0.139	NA^2^	41.9 ± 6.8	44.4 ± 8.0	2.5 (−1.3–6.3)	11	0.128	NA^2^

1. Range 0–100; higher scores means better ability to participate or more satisfaction. 2. Not analysed as < 30 patients in this diagnostic category. Sample size too small to calculate effect size. 3 Not analysed as no statistically significant change score (T1 vs T0). *Abbreviations*: *PROMIS-APS* Ability to Participate in Social Roles and Activities, *PROMIS-SPS* Satisfaction with Social Roles and Activities;T0, start outpatient rehabilitation; T1 end outpatient rehabilitation, *CI* Confidence Interval, *NA* not analysed

Highest scores for both APS and SPS at admission were seen in the amputation group (44.0 ± 7.9 and 42.6 ± 8.1, *n* = 29) and lowest in the musculoskeletal disorder group (41.4 ± 6.8 and 40.9 ± 7.5, *n* = 81).

## Discussion

We found small improvements of participation as assessed with the PROMIS-APS and PROMIS-SPS after rehabilitation. In our study, the PROMIS-APS and PROMIS-SPS short forms were completed online by more than half of the patients and about two thirds at discharge, without sending reminders. The finding that lower levels of participation were found at the start of outpatient rehabilitation may indicate that referring physicians and rehabilitation physicians were able to make an appropriate selection of patients for rehabilitation.

Although at discharge the observed levels were still lower than those of the general population, for both the PROMIS-APS and the PROMIS-SPS improvements were seen over time. Improvements of about 2 to 6 T-score points in PROMIS measures have been found to be minimally important to patients [20, 21], which means that improvements lower than 2 to 6 are on average not perceived as important improvement. Taking into account the effect size observed in the present study, in previous research, in a comparable patient group, comparable improvements were found with the Utrecht Scale for Evaluation of Rehabilitation-Participation (USER-P) subscale ‘satisfaction’ (effect size 0.31 vs 0.36) with the PROMIS-SPS [22]. However, larger changes were found with the USER-P subscale ‘restrictions’ compared to the PROMIS-APS (effect size 0.49 vs 0.16) [22]. These differences in effect sizes might be explained by the fact that the USER-P subscale ‘restrictions’ consists of items that are slightly more related to limitations of activities of daily living rather than participation in comparison to the PROMIS-APS. Outpatient rehabilitation may have more impact on limitations in daily living than participation. This does however not necessarily mean that the effect of rehabilitation is smaller or absent. It may be related to the fact that participation is a complex construct, which is influenced by many aspects, such as economic factors or social support. It may thus appear that it is easier to demonstrate the effect of rehabilitation within the domain impairments than participation [23]. Moreover, the patients’ perception of participation may change over time. In particular when the rehabilitation phase ends and patients are returning to their life situations, they may face challenges that increase with the number of roles patients are willing and/or able to take up or that patients became more aware of their participation restrictions.

Another possible explanation for the small improvements on the PROMIS-APS might be that improving participation may take more time than spent in outpatient rehabilitation. On the other hand, it is also possible that due to outpatient rehabilitation, patients became more aware of their participation restrictions. We thus need measurement instruments that are flexible to adjust to individual participation goals changing or emerging with time, it is clear that the (ecological) validity of the PROMIS-APS and PROMIS-SPS in rehabilitation warrants further research.

Although very relevant for clinical practice, it is difficult to draw conclusions on the extent to which rehabilitation actually has an impact on participation from the results of our study. Either the observed small changes on PROMIS-APS and PROMIS-SPS reflect true small changes in participation or the PROMIS-APS and PROMIS-SPS are insufficiently able to measure changes in participation. In the absence of a gold standard to reflect changes in participation a study including multiple other measures of participation would be needed to answer this question.

In this study, we used the PROMIS-APS and PROMIS-SPS short forms. We do however, recommend to use the PROMIS CATs in future research, as the CATs measure participation more precisely than the short forms [10]. A personalised PROMIS short form could also be considered, e.g. a short form consisting of the five most problematic items for the patient at issue, as rehabilitation treatment is focused on patient’s request for help and, therefore, tailor-made. The use of tailor-made short forms appeared to be feasible [24].

### Study limitations

Despite the overall good response, fewer patients completed the forms at discharge as compared to admission. As patients who completed both measurements differed from those with one measurement, selection bias might hamper the generalisability of our findings. In particular, the number of patients at discharge were too low to calculate the effect size for four of the diagnostic categories. To improve the response rates we recommend to send reminders, and provide patients and healthcare professionals with feedback on their scores [25].

Another limitation is that it was not possible to determine responsiveness and interpretability of the PROMIS-SPS and PROMIS-APS as we did not include comparator instruments or an anchor question at follow-up. We therefore recommend for further research to study the responsiveness and interpretability of the PROMIS-SPS and PROMIS-APS and identify the Minimal Important Change using an anchor-based method.

## Conclusions

Patients in outpatient rehabilitation have considerably lower levels of societal participation as compared to the general Dutch population, and show small improvements at discharge.

## Abbreviations

ICF: International Classification of Functioning
WHO: World Health Organisation
IRT: Item Response Theory
CAT: Computer Adaptive Test
PROMIS: Patient-Reported Outcomes Measurement Information System
PROMIS-APS: Ability to Participate in Social Roles and Activities
PROMIS-SPS: Satisfaction with Social Roles and Activities
PRO: Patient-reported outcomes
SD: Standard Deviation
USER-P: Utrecht Scale for Evaluation of Rehabilitation – Participation
